# Origin of Shear Stability and Compressive Ductility Enhancement of Metallic Glasses by Metal Coating

**DOI:** 10.1038/srep27852

**Published:** 2016-06-08

**Authors:** B. A. Sun, S. H. Chen, Y. M. Lu, Z. G. Zhu, Y. L. Zhao, Y. Yang, K. C. Chan, C. T. Liu

**Affiliations:** 1 Centre For Advanced Structural Materials, Department of Mechanical Biomedical Engineering, City University of Hong Kong, Hong Kong; 2 Advanced Manufacturing Technology Research Centre, Department of Industrial and Systems Engineering, The Hong Kong Polytechnic University, Kowloon, Hong Kong; 3 Department of Physics and Materials Science, City University of Hong Kong, Hong Kong

## Abstract

Metallic glasses (MGs) are notorious for the poor macroscopic ductility and to overcome the weakness various intrinsic and extrinsic strategies have been proposed in past decades. Among them, the metal coating is regarded as a flexible and facile approach, yet the physical origin is poorly understood due to the complex nature of shear banding process. Here, we studied the origin of ductile enhancement in the Cu-coating both experimentally and theoretically. By examining serrated shear events and their stability of MGs, we revealed that the thin coating layer plays a key role in stopping the final catastrophic failure of MGs by slowing down shear band dynamics and thus retarding its attainment to a critical instable state. The mechanical analysis on interplay between the coating layer and shear banding process showed the enhanced shear stability mainly comes from the lateral tension of coating layer induced by the surface shear step and the bonding between the coating layer and MGs rather than the layer thickness is found to play a key role in contributing to the shear stability.

At temperatures far below the glass transition, metallic glasses (MGs) are susceptible to shear localization with plastic strain highly concentrated in narrow regions or shear bands^1^,^2^,^3^,^4^. Although not unique to MGs, shear bands have importance consequences on microscopical mechanical behavior of MGs. On one hand, owing to the significant strain softening^5^,^6^,^7^,^8^,^9^,^10^, a shear band has high propensity for runaway propagation, especially under the tensile loading^11^. This results in the macroscopical brittleness of MGs and has severely limited their structural applications^12^,^13^,^14^. On the other hand, proliferation of multiple “stable” shear bands^15^,^16^,^17^,^18^ could largely increase the ductility of MGs, since these bands offer the much larger volume to accommodate the plastic strain that is supposed to be sustained by a single shear band. Over past decades, many effective approaches have been proposed to improve the ductility of MGs. These includes internal composition/structure modification^19^,^20^,^21^, *in-situ* or *ex-situ* introduction of crystalline phases^22^,^23^,^24^,^25^, surface residual stress control^26^,^27^,^28^, nanolamination^29^,^30^, geometrical constraint^31^,^32^,^33^ and so on, strategies all based on the principle of shear band stabilization and proliferation. Despite the progresses, engineering shear bands in a controllable and desirable manner in MGs remains a crucial task, due to the lack of a thorough understanding on the shear banding process and its interplay with various intrinsic and extrinsic factors^2^.

Among extrinsic toughen strategies, metal (such as Cu and Ni) coating by electroplating^31^,^33^,^34^,^35^,^36^ is regarded as an flexible and facile approach to improve the plasticity of MGs. For example, it was reported^31^ that a Cu layer with a thickness of only a few tens of micrometers electroplating on the surface of a Zr-based MG could readily increase the plasticity from 1% to 5%. In addition, the coating thickness can be easily controlled by tuning plating time and current, and meanwhile, the electroplating will not cause damage on the surface of MGs^33^. In general, the mechanism for the plasticity enhancement can be attributed to the confinement effect of coating layer on the shear band propagation during deformation. However, as compared to other geometrical constraint methods such as shrink-fit metal sleeve in mm-scale^32^, the inhibit of shear band propagation by the coating layer is not so evident, considering the layer’s small thickness (in the micrometer range) and low yield strength (~100 MPa for deposited Cu^37^) as compared to those of MGs the layer electroplated on. In view of this, it is difficult to understand why the coating layer is so effective to enhance the plasticity of MGs. While some mechanisms^31^,^33^,^35^ were proposed, such as the “crack buffer zone” model^33^, a quantitative understanding on the interplay between the coating layer and the shear banding process is still lacking.

To understand the underlying mechanism for the plasticity enhancement by coating, we noticed that the shear banding in the compression of MGs often proceeds in an intermittent or stick-slip manner, which manifested as the serrated flow behavior^38^,^39^,^40^ in stress-strain/time curves. As in a serrated event, the shear band motion can be arrested after reaching a maximum velocity, different from the runaway shear banding during the final fracture, serrated flow is often regarded as “stable” shear banding process^41^ and closely correlates with the plasticity of MGs. Since only a small portion of elastic energy stored in MG is released during a serrated event, the typical stress drop for serrations is around a few tens of MPa^42^, which is much smaller as compared to the strength of MGs (usually larger than 1500 MPa for Zr-based MGs) , yet in the same order of magnitude of the yield strength of coating layer. Therefore, it is expected that the coating layer will have a significant effect on the shear band dynamics during serrated flow, and consequently affect the shear stability or plasticity of MGs. In this work, we conducted a systematical investigation of the effect of Cu-layer coating on the serrated flow and shear banding process under various conditions, in an attempt to extract the underlying mechanism for plasticity enhancement. It was found that the thin coating layer plays a vital important role in stopping the runaway propagation of a shear band for fracture, by slowing down shear band dynamics, thus retard its attainment to a critical instable state. A quantitative mechanical analysis on the interplay between the coating layer and shear banding dynamics was also presented to reveal the physical origin of shear stability enhancement by the metal coating.

## Results

### Deformation behavior of as-cast and coated MGs

**Figure 1:**
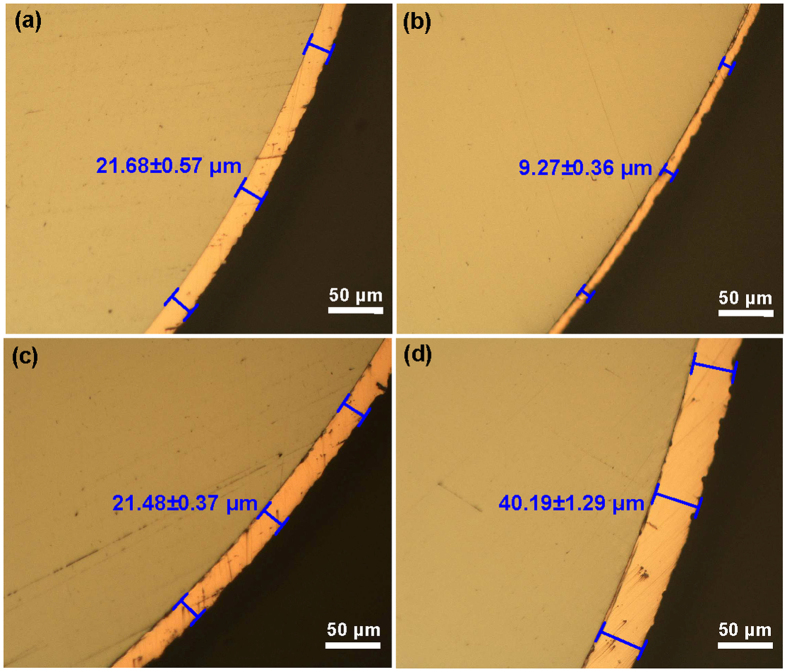
The optical micrographs for coated Vit105 MGs, from which the coating layer thickness were measured: (**a**) D = 2 mm coated for 45 min; (**b**) D = 3 mm, coated for 15 min; (**c**) D = 3 mm, coated for 45 min; (**d**) D = 3 mm, coated for 90 min.

Figure 1 shows the cross section of coated samples with a diameter of 2 mm and 3 mm for Vit105 MG. The MG samples for D = 2 mm (D is the diameter) were plated for about 45 min, while the samples for D = 3 mm were plated for different times (15 min, 45 min and 90 min), in order to investigate the effect of coating thickness on the plastic deformation behavior of MGs. As can be seen from Fig. 1, the average thickness of Cu-layer have an average thickness of 9.27 μm for samples (D = 3 mm) coated for 15 min, and increases to 21.48 μm and 40.20 μm for those coated for 45 min and 90 min, respectively. The samples of 2 mm in diameter coated for 45 min also have a Cu-layer thickness of 21.68 μm. It seems that the coating thickness is mainly determined by the plating time and weakly dependent on the sample diameter. The average coating thickness for all samples were measured and summarized in Table 1.

**Figure 2:**
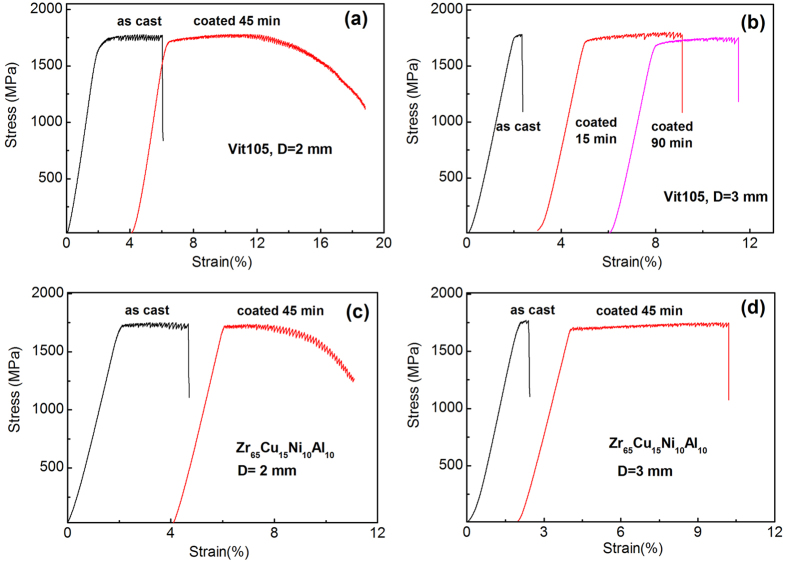
The engineering stress-strain curves for as-cast and coated MG samples tested at a constant strain rate 5 × 10^−4^ s^−1^: (**a**) Vit105, D = 2 mm; (**b**) Vit105, D = 3 mm; (**c**) Zr_65_Cu_15_Ni_10_Al_10_, D = 2 mm; (**d**) Zr_65_Cu_15_Ni_10_Al_10_, D = 2 mm.

Figure 2 shows typical stress-strain curves for various MG samples with different compositions, sizes and coating thicknesses. One can see from the figure that all samples yield at about 2%, and then display different plastic strain before the final failure. Obviously, the plasticity or shear stability for all MGs is significantly enhanced by the Cu-layer coating, as evidenced by the increased final plastic strain. For example, the as-cast Vit 105 MG (D = 2 mm) shows only a plastic strain of ~3.9%, and then fails catastrophically along the dominant shear plane. In contrast, the sample coated with a Cu-layer in the same composition and size could displays at least a plastic strain of 10%, and meanwhile the sample doesn’t fails abruptly, but proceeds with a gradual decreasing stress at the final deformation stage [see Fig. 2(a)]. The similar case also holds for Zr_65_Cu_15_Ni_10_Al_10_ MG samples (D = 2 mm). While for MGs with *D* = 3 mm, both the as-cast and coated samples failed in a catastrophic manner. However, the coated samples still display impressive plasticity [see Fig. 2(b,d)], as compared with those as-cast ones which display almost negligible plasticity after yielding. It is worth noting here that there seems no direct correlation between the plasticity improvement and the coating thickness, which defies the conventional notion that the thicker coating layer leads to the larger plasticity improvement^33^,^34^. As can be seen from Fig. 2(b), the sample with the least thick coating layer (~9 μm) displays the even larger plasticity than the sample with a thick coating layer (~40 μm). The reason can be partially attributed to the fact that the coating thickness varies in a small range in our work. On a more profound level, however, the reason lies that the coating thickness within some range is not the controlling factor for the plasticity enhancement, which will be discussed in Section 4.2 in detail. In addition, the yield strength of coated MGs is slightly lower than that of as-cast samples, as listed in Table 1. The strength reduction is in general proportional to the coating thickness. This is understandable considering the low strength of electroplated Cu-layer (100–150 MPa)^37^ compared to that of MGs.

### The effect of coating on serrated flow and shear banding

**Figure 3:**
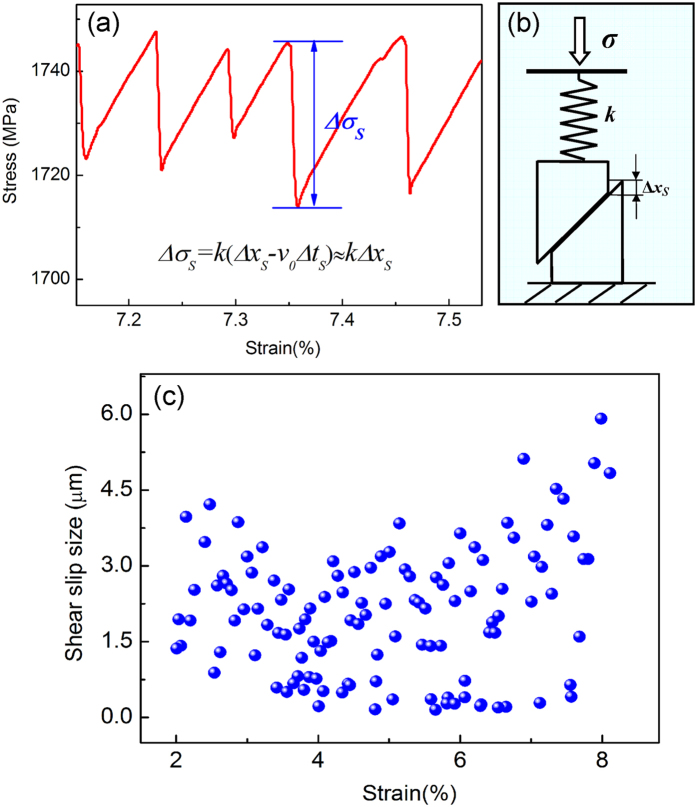
(**a**) A segment of stress-strain curve (coated Zr_65_Cu_15_Ni_10_Al_10_, D = 3 mm) shows typical serrations. For each serration, the stress drop amplitude can be determined. (**b**) The schematic diagram for the stick-slip model of a single shear band in MGs. (**c**) The variation of shear slip size with the deformation strain for a typical MG sample (coated Zr_65_Cu_15_Ni_10_Al_10_, D = 3 mm).

After entering into the plastic deformation regime, both as-cast and coated MGs displays obvious serrated flow behavior, which manifests as repeated cycles of a sudden stress drop followed by an elastic reloading part [Fig. 3(a)]. As shown by recent studies, serrated flow in compression of MGs reflects the stick-slip or intermittent shear banding process^43^. Specifically, the elastic loading part in the serration corresponds to the “stick” phase of shear banding, where the band moves in a velocity much slower than the loading rate (*v* ≪ *v*_*0*_), while the sudden stress drop part corresponds to the rapid shear band operation with the sliding velocity is much larger than the loading rate (*v* ≪ *v*_*0*_)^40^,^44^. During the “slip” phase, the shear band sliding could partially release the elastic energy stored in the machine-sample system (MSS)^45^. According to the stick-slip model of shear banding [see Fig. 3(b)], the vertical shear-band slip size Δ*x*_*S*_ in during a serrated event relates with the stress drop magnitude Δ*σ*_*s*_ by^40^


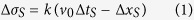


where *v*_*0*_ is the loading rate, Δ*t*_*s*_ is time duration of the shearing event, *k* is the elastic constant of MSS. *k c*an be calculated according to the equation^41^: *k* = *E*/(*H* + *πED*^2^/4*κ*_*M*_) with *E* and *H* being Young’s modulus and height of the sample, respectively, and *κ*_*M*_ the machine stiffness, all of which can be experimentally measured. Since during a shearing event, the shear band slides at a much larger velocity than the loading rate, i.e., *v*_*S*_ ≫ *v*_*0*_, we have *v*_*0*_Δ*t*_*S*_ ≪ Δ*x*_*S*_, and Eq. 1 becomes Δ*σ*_*S*_ ≈ *k*Δ*x*_*S*_. Therefore, one can obtain Δ*x*_*S*_ ≈ Δ*σ*_*S*_/*k*. The shear band slip size during a serrated event can be obtained if the stress drop magnitude and other parameters (*E, D, H* and *κ*_*M*_) are measured.

With the approach above, we could determine the stress drop magnitude and then calculate the shear slip size for all serrations in a single deformation curve of MGs, as can be seen from Fig. 3(c) for the typical MG sample (coated Zr_65_Cu_15_Ni_10_Al_10_, D = 3 mm). Among all shear slip sizes in a single deformation curve, we could extract a maximum value 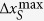, which usually appears around the final fracture, as can be seen from Fig. 3(c). Since 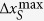 is the largest shear slip size that a shear band undergoes before the final fracture, it is reasonable to consider the 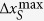 as the critical shear slip size beyond which shear band instability occurs. At the critical state, cracks quickly forms and propagate in the shear band, and results in the final failure of MGs. The 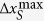 for all MG samples as listed in Table 1 is thus determined. One can see that 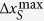 is almost a constant value (5.56 ± 0.28 μm) except for the as-cast 3 mm-diameter MGs where only a few serrations are recorded before the final fracture. This constant 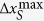 for MGs under various conditions seems suggested that the instable or runaway shear banding is controlled by a critical shear slip size beyond which final failure of MGs occurs. Considering the measured time duration for serrated events is in the order of ~1 ms, the constant critical shear slip size observed for various MGs may corresponds to a critical shear band velocity for the failure of MGs, which may rooted in the liquid-type instability of shear banding process^46^. In this sense, the constant critical shear slip sizes for the as-cast and coated MGs seems suggest that the coating layer plays a key role in resisting to the runaway shear band propagation.

**Figure 4:**
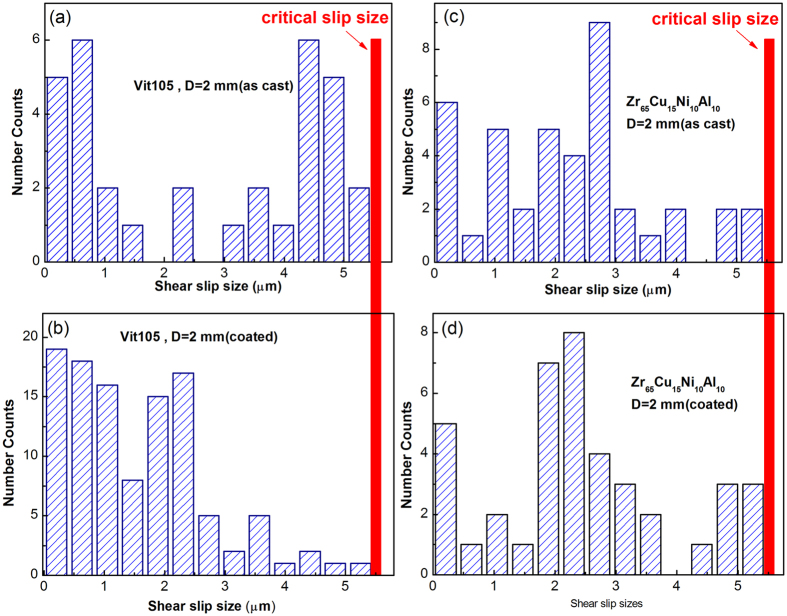
The number distribution histograms of shear slip size for 2 mm-diameter MG samples: (**a**) Vit105, as cast; (**b**) Vit105, coated for 45 min; (**c**) Zr_65_Cu_15_Ni_10_Al_10_, as cast; (**d**) Zr_65_Cu_15_Ni_10_Al_10_, coated for 45 min. All histograms are terminated almost at the same critical shear slip size (the red line).

We also performed a statistical analysis on the distribution of shear slip sizes, which could provide important information on shear banding dynamics. Since the measured resolution for stress drops for our experimental setup is around 1 MPa, shear slip sizes induced by serrations with a stress drop less than 1 MPa were not counted in our statistics. Figure 4 show typical number distribution histogram of shear slip sizes both for as-cast and coated MGs (Vit105 and Zr_65_Cu_15_Ni_10_Al_10_, D = 2 mm). One can see that all distribution histograms exhibit a peak-like shape except at initial small Δ*x*_*S*_. This indicates that a characteristic length scale exists for serrated events induced by shear band slipping. As has been reported recently^16^,^47^, this kind of distribution may corresponds to the chaotic stick-slip dynamics, which is mainly associated with the single dominant shear band behavior where only a few state variables (strain rate, free volume, temperature *etc*)^47^ interplay with each other during deformation process. This is different from the power-law distribution with no characteristic scales as observed in some ductile MGs^16^,^17^, which is associated with collective dynamics of multiple shear bands. For the peak-like distribution, the average or characteristic shear slip size, 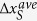, can be determined for each histogram. One can clearly see that the coating samples have the smaller 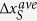 than the as-cast ones. For example, the 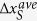 for the coated Vit105 sample (D = 2 mm) is around 1.36 μm, which is just more than half of that of the as-cast one (2.39 μm). The smaller 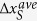 often indicates a more “stable” shear banding, since it has less chance to fluctuate to the critical 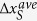 for shear-band instability (the red line that histograms terminate in Fig. 4). Similar statistical results are also obtained from the samples with D = 3 mm, as listed in Table 1. Based on these results, we can infer that although the coating layer plays a trivial effect in stopping the runway shear band propagation, it could effectively stabilize the shear banding process by slowing down its dynamics, thus retards its attainment to the critical instable state.

### The deformation morphology

**Figure 5:**
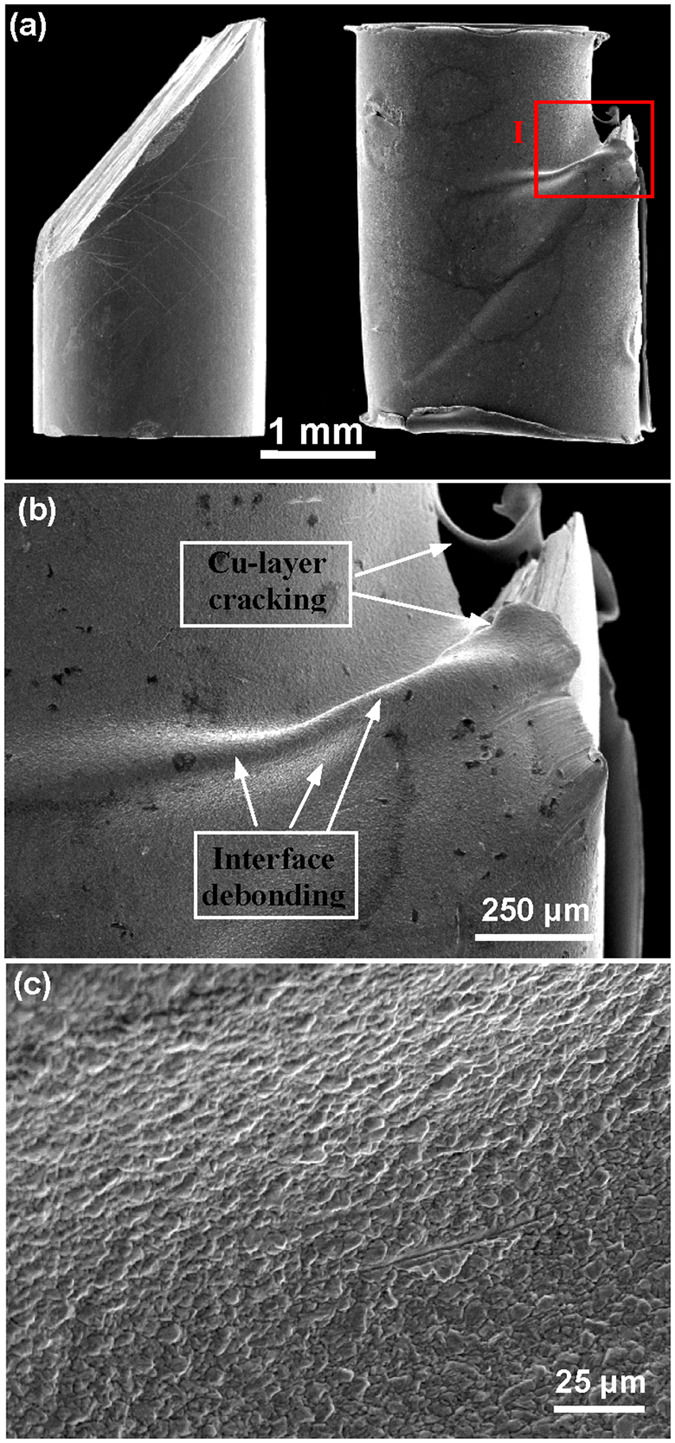
Deformation morphology for as-cast and coated MGs (Vit105, D = 2 mm). (**a**) The SEM micrographs showing the side view for the as-cast sample and the coated sample (the right) after final failure. (**b**) An enlarged view around the shear step for the coated sample, showing the cracking of coating layer and the interface debonding. (**c**) An enlarged view on the region of debonding, showing features for severe plastic deformation of the coating layer.

**Figure 6:**
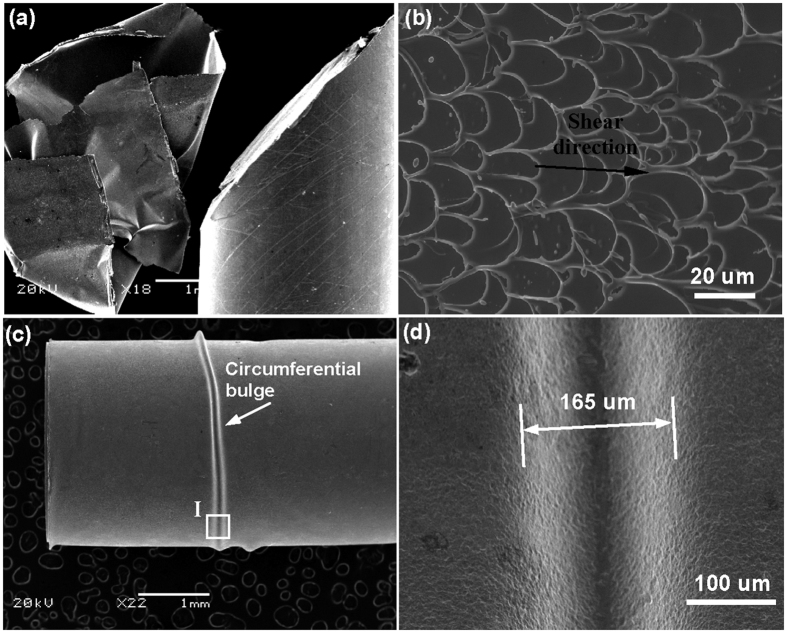
Deformation morphology for coated MGs (Zr_65_Cu_15_Ni_10_Al_10_, D = 3 mm, coated for 45 min). (**a**) The SEM micrographs showing the fractured coated sample and the coating layer is completely peered off. (**b**) The formation of vein-like patterns on the fracture surface. (**c**) The morphology for the coated sample compressed to a plastic strain around 2%, showing an obvious circumferential bulge. (**d**) The enlarged view for the bulge, showing features for severe plastic deformation. The width for the bulge measured is around 165 μm.

To reveal more details for the effect of coating layer on the deformation of MGs, we examined the deformation and fracture morphologies of both as-cast and coated MGs. As can be seen from Fig. 5, the coated 2 mm-diameter MGs do not fails catastrophically after deformation [see Fig. 2(a)], but proceeds with the gradually decreasing stress, in contrast to as-cast ones, which fails catastrophically along one dominant shear plane. In the later deformation stage, the coating layer has been pierced by the tip of surface shear step of shear banding along the dominant shear plane, as can be seen from Fig. 6(b). The further shearing will induce the cracking or breaking up of Cu-layer along the lateral direction, which extends from the shear step tip to the sample end. This indicates that the cracking of coating Cu-layer and the plastic deformation zone in the crack tip is one effective way to dissipate the released elastic energy in the later deformation stage. In addition, we also observed obvious bulges on the coating layer in the region near the surface shear step [see Fig. 5(b)]. The formation of bulges should be associated with the debonding of coating layer from MG surface, which may be attributed to the deformation mismatch between coating layer and MG sample. An enlarged view of a bulge [shown in Fig. 5(c)] shows many scaly patterns in micro-scales on the coating layer, which indicates that severe plastic deformation of coating layer has occurred before interface debonding.

**Figure 7:**
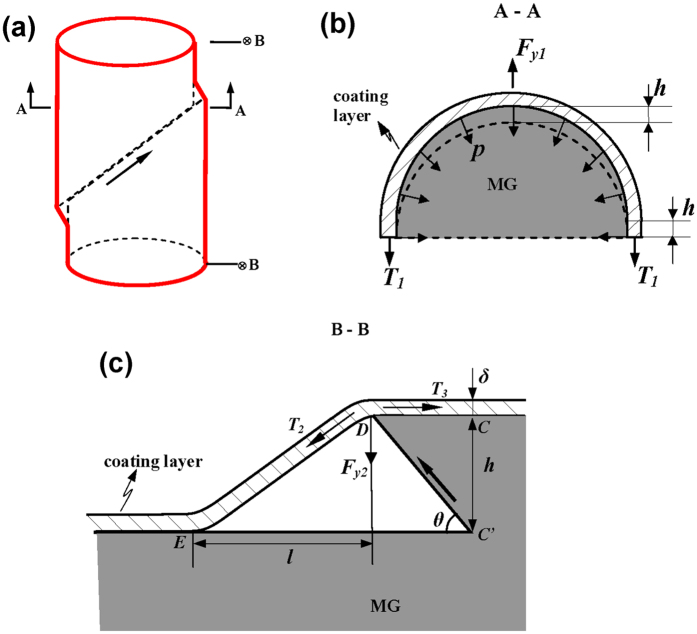
Schematic illustration for the interplay between the coating layer and the shear banding during MG deformation: (**a**) The overall view for the confinement of the coating layer on the MG. (**b**) The cross-section view showing the circumferential tension of the coating layer. (**c**) The longitudinal section showing the lateral tension of the coating layer.

The case is slightly different for the 3 mm-diameter MG samples. Both as-cast and coated samples fail catastrophically after some plastic strain (even with different plastic strains). Similar with the as-cast sample, the coated sample also fails catastrophically along the dominant shear plane and vein-like patterns^18^ can be observed on the fracture surface of coated MGs. After the fracture, the coating layer was completely peeled off from the sample after fracture [see Fig. 6(a)]. This may be due to the elastic energy that is released in the final fracture is so large to result in complete debonding of the coating layer. (This is the main reason that 3 mm samples failed catastrophically.) Many fine secondary shear bands are also observed on the lateral surface of coated MG sample, in consistent with the improved plasticity compared to as-cast samples. We also stopped compression tests deliberately to observe deformation morphology of coated MGs. As shown in Fig. 6(c), one can also see a circumferential bulge formed on the coating layer. The bulge, with a width of about 160 μm, is initiated near the shear step formed by the dominant shear plane and almost makes a circle on the lateral surface of the MG. As can be seen from Fig. 7(d), features for severe plastic deformation were also observed near or on the surface of bulge. The formation of bulge should also be associated with the debonding of the coating layer due to the tension exerted by the surface shear steps due to shear band sliding.

## Discussion

**Figure 8:**
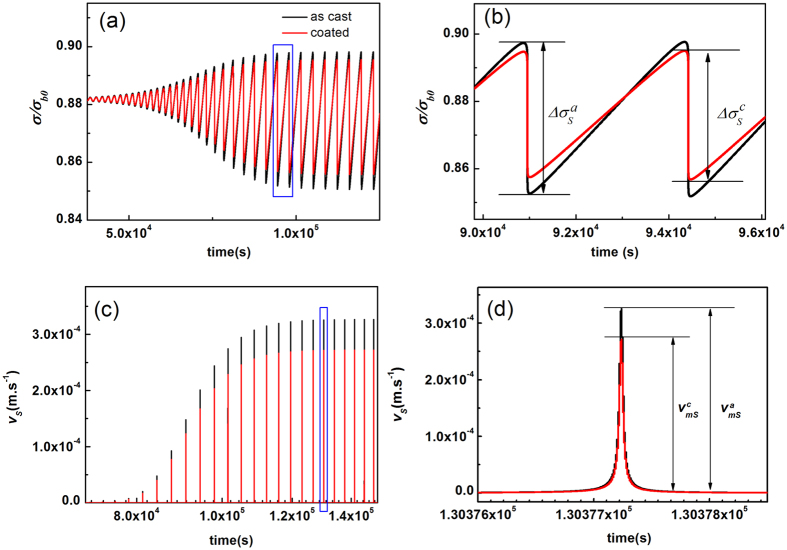
Numerical calculations showing the slowing downing stick-slip shear dynamics by the presence of coating layer. The calculation details (the dynamics equation used, the values of various parameter used as well as the integration method) can be found in ref. 43. The *k* is chosen as *k* = 0.8 *k*_*cr*_, and the *k*_*r*_induced by the coating layer is chosen as *k*_*r*_ = 0.2 *k*. (**a**) The calculated serrated stress-time curves for the as-cast sample (black line) and the coated samples (red line), respectively. (**b**) The enlarged view for the rectangular region in (**a**), showing that the reduced stress drop magnitude (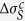) for the coated sample compared to that of the as-cast sample (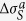), and 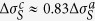. (**c**). The calculated shear-band velocity profile for the as-cast sample (black line) and the coated samples (red line), respectively. (**d**) The enlarged view for the region in (**c**), showing that the maximum shear band velocity (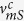) during a serrated event is reduced by the coating layer, and 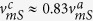, 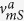 is the maximum shear band velocity of the as-cast sample.

From results above, one can see that the coating layer mainly interacts with the shear banding process during the deformation by applying the confinement effect on the surface shear steps. From the viewpoint of material science, the interaction between the coating layer and MGs could be affected by many factors, such as the chemistry of the Cu/MG interface, the grain structure and size of the coating layer, etc. These factors result in different interface bonding and mechanical response of the coating layer during deformation, which are difficult to track. For the sake of simplicity, we would like to tackle this issue by analyzing the mechanical stress arising from the interaction between the coating layer and MG during deformation. Based on this mechanistic approach, various aspects of the effects of the coating layer on the shear stability of MGs can be quantitatively studied. Figure 7 shows schematically for the confinement of the coating layer on the deformation of MGs. Once a shear step slides out of the surface of MGs by shear banding, the coating layer near the shear step will be subjected to a uniform tension along circumferential direction and a lateral tension, respectively. Conversely, the coating layer imposes transverse forces *F*_*y1*_ and *F*_*y2*_ to resist to the shear band sliding during deformation, as shown in Fig. 8. *F*_*y1*_ and *F*_*y2*_ are induced by the circumferential tension and the lateral tension of the coating layer, respectively. As shown in Supplementary Note I, *F*_*y1*_ and *F*_*y2*_ can be expressed as:


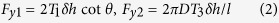


where *T*_*1*_ and *T*_*3*_ are the circumferential tension stress and lateral tension stress, respectively, *δ* is the coating-layer thickness, *h* is the height of surface shear step, *θ* is the shear angle and *l* is the is lateral length projected by the bent part of coating layer and (*l* + *h* cot *θ*) can be regarded as the debonding length (See Fig. 7). *T*_*2*_ and *T*_*3*_ initially increase with *h* and quickly reach the yield strength of Cu-layer and after then, increase slowly until they reach tensile fracture strength 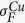, where the fracture of coating layer may occur.

With the increase of *h*, debonding of the coating layer along sample lateral may also occurs, which release the elastic energy stored in the coating layer and result in the increase of *l*. We also present a quantitative analysis on the critical condition for the occurrence of debonding (see Supplementary Note II). The results show that the debonding is associated with a critical ratio of *h/l*, *λ*_*c*,_which is deterimined by the interface toughness *Γ*_*i*_. Given a certain *h*, debonding occurs for *l* < *l*_*c*_ = *h*/*λ*_*c*._Similarly, for a certain *l*, debonding occurs for *h* > *h*_*c*_ = *lλ*_*c*._. At the beginning when the shear step is just slides out of surface, *l ≪ h* and *h/l* should be much larger than *λ*_*c*_, interface debonding has to occur to release the stored elastic energy in the coating layer, otherwise, the tension stress *T*_*2*_ will be so large to cause the fracture of the coating layer. However, once a stable length *l* is created, the coating layer could either be deformed with increase of the tension stress until the final fracture or debond further with the increase of *l* and meanwhile maintaining or decreasing the tensile stress, which depends on the specific coating quality or interface toughness. The lower *Γ*_*i*_ often leads to debonding rather than fracture of the coating layer, which will not be effective to resist to shear band sliding, as will be shown below.

With the stress analysis above, we are now at the position to analyze the mechanism for retarding shear band dynamics by the coating layer. The coating layer imposes a transverse force (*F*_*y*_ = *F*_*y1*_ + *F*_*y2*,_) to the MG to resist to the shear band sliding during deformation. If we simply neglect the pressure dependence of shear resistance, applying *F*_*y*_ will increase the shear resistance (in vertical direction) of the shear band by:


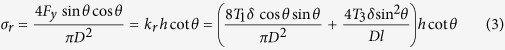


where *k*_*r*_ is defined by the terms in the bracket in the right of Eq. 5. Now, the dynamic equation for the dominant shear band^40^,^43^ in the presence of coating layer is written as:


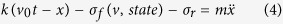


Considering that the vertical shear displacement *x* = *h* cot *θ*, Eq. 6 becomes:


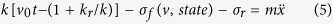


where *σ*_*f*_ is the resistance stress of shear band, which is a function of the shear band velocity *v* (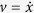) and the internal state variable (such as the free volume content) of shear band. By further assuming the specific state variable and its evolution dynamic equation, Eq. 5 can be well solved as typical stick-slip shear band motions, which results in the periodic serrations in the stress-strain/time curves^41^,^43^. Compared to the dynamic equation of shear band without the coating layer, one can see that the coefficient of shear displacement *x* is increased by the factor (1 + *k*_*r*_*/k*). This means that, if the shear slip size for serrated events is Δ*x*_*S*_ for an as-cast sample, the shear slip size by coating a layer on it will be reduced to Δ*x*_*S*_/(1 + *k*_*r*_/*k*), provided that the test conditions are the same for the as-cast and coated samples. This also holds for the stress drop amplitude Δ*σ*_*S*_ (Δ*σ*_*S*_ = *k*Δ*x*_*S*_) and the shear band velocity *v*_*S*_ (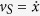), which are all reduced by the factor (1 + *k*_*r*_/*k*). These changes on shear-band dynamic characteristics can be also seen in Fig. 8, where the stick-slip motion of a single shear band is numerically solved with the effective disorder temperature as the internal state variable. Therefore, the coating layer could effectively improve the shear banding stability by slowing down its stick-slip dynamics, if the magnitude of *k*_*r*_ is comparable to that of *k*. However, as the stress (*σ*_*r*_) that the coating layer exerts to the MG is quite small as compared to the fracture strength of MG, it seems that the coating layer plays a trivial role once the final catastrophic fracture occurs.

From Eq. 3, one can see that *k*_*r*_ comprises of two terms, which are related with the circumferential tension and lateral tension of the coating layer, respectively. For simplicity we denoted them as *k*_*r1*_ [*k*_r1_ = 8*T*_1_*δ*cos *θ* sin *θ* /(*πD*^2^)] and *k*_*r2*_ [*k*_r1_ = 4*T*_3_*δ* sin^2^ *θ*/(*Dl*)], respectively. With these expressions, the magnitude of *k*_*r1*_ and *k*_*r2*_ can be estimated. By taking *T*_*1*_ = *T*_*3*_ ~ 100 MPa, *δ* ~ 20 μm, *D* ~ 2 mm, *θ* ~ 45°, *l* ~ 5 μm in the order of shear step height *h*, it is estimated that *k*_*r1*_ ~ 0.6 GPa.m^−1^, *k*_*r2*_ ~ 800 GPa. m^−1^. While the value of *k*_*r1*_ is very small compared to that of *k* (6000~10000 GPa.m^−1^) for typical MGs^43^ in compression, the value of *k*_*r2*_ is well exceeds one tenth of the lower bound of *k*. This means that the average shear band slip size and sliding velocity in a shearing event can be reduced by 10% due to the lateral tension of the coating layer, and this could well enhance the shear band instability and thus improve the plasticity. From the derivation of *k*_*r2*_, it seems that the debonding length *l*, rather than the coating thickness, play the key role in the shear stability enhancement. In fact, the importance of interface/surface states on the shear stability of MGs has long been recognized in the *in-situ* TEM studies of MGs^48^, where the nanoscale surface modulations associated with local deformation domains of a nanometer size are either cavities or hillocks and depending on size and aspect ratios surface/interface state is very critical for the shear instability. This explains the fact that the plasticity improvement has no direction relation with the coating thickness as observed in our experiments. As discussed in Section 4.1, *l* is mainly determined by the interface toughness *Γ*_*i*_. The larger *Γ*_*i*_, corresponding to the smaller *l*, results in the larger *k*_*r2*_, and thus is beneficial for the plasticity improvement. In this sense, the bonding between the coating layer and MG surface has to be considered in designing the mechanical behavior and plasticity of MGs by the coating method. However, the debonding length *l* is also dependent on the coating layer thickness. As can be seen from Supplementary Note II, with the increase of the coating layer thickness, the critical ratio *λ*_*c*_ also decreases, means that the thick layer are more susceptible to debonding, resulting in a large *l*. For some critical value, the coating thickness may play a dominant role over the interface bonding in enhancing the shear stability of MGs. This may explains experimental observations that the plasticity improvement positively correlates with the coating layer thickness^49^. In other words, one has to bear in mind that the dominance of interface bonding in the ductility improvement of MGs is only applied to thin coating layers (less than 50 μm in general). It is noted that for a given *Γ*_*i*_, *l* should increase with the shear step height *h* during the deformation process. Thus, the shear-stability enhancement by the coating is the most effective at initial deformation stage when *l* is small (in the order of a few micrometers). As the deformation proceeds, the stability enhancement is largely weakened with the increase of *l* and the decrease of *k*_*r2*_, particularly at the final deformation stage (*l* ~ 100 μm in order of the bulge width).

The discussion above on the mechanism of ductility enhancement is limited to the framework of the stability of the single dominant shear band. Along with the enhanced shear stability of the dominant shear bands, multiple shear bands may also be induced to accommodate the external applied strain and these bands could also contribute to the plasticity improvement of coated MGs. If these secondary shear bands could slide out of the MG surface, they are also interact with the coating layer and the effects of the coating layer on the dynamics of multiple shear bands could also be analyzed similarly by considering an interaction term in the dynamic equations^40^. In addition, previous studies^50^,^51^,^52^ also showed that sample aspect ratio and stress state also play an important role in the shear stability of MGs. We performed compression tests on as-cast and coated MGs with different aspect ratios (results unpublished), which showed that the effect of significant plasticity enhancement by the coating layer only appears for some intermediate range of aspect ratios (around 2:1). At lower aspect ratios, both as-cast and coated samples become barreled with the formation of multiple shear bands and even are compressed into thin discs (the aspect ratio lower than 1:1). While for the high aspect ratio (e.g. 3:1), both as-cast and coated samples fail catastrophically with litter plasticity. These results clearly indicate that the main mechanism for the plasticity enhancement by metal coating arises from the interplay between the stick-slip dynamics of the dominant shear band and the coating layer. Also, the mechanism for the ductility enhancement could not be applied to the tension or bending of MGs where the stable stick-slip shear banding process is absent. The details for the effects of metal coating on the deformation behavior of MGs and the underlying mechanisms in these cases deserve a further study.

In summary, we systematically investigate the effect of the softening metal coating on the plastic deformation behavior of MGs by varying their composition, size and coating thickness. Under all conditions, we found that the shear instability of MGs occurs at an almost constant critical shear-band slip size during serrated flow, while the coating layers results in the smaller serrations and average slip size. Combined with observations on the deformation and fracture morphology, it seems that the thin soft metal coating layer plays a key role in stopping the final catastrophic fracture of MGs by slowing down shear band dynamics, thus enhance the shear stability and plasticity of MGs. The quantitative stress analysis on interplay between the coating layer and shear banding process showed that the enhanced shear stability mainly comes from the lateral tension of coating layer induced by the surface shear step. The interface bonding between the coating layer and MGs rather than the layer thickness, is found to play a key role in the shear stability enhancement. These results are helpful to control and design the plasticity of MG by coating method and provide important insights on physical nature of shear stability in MGs.

## Methods

Two MGs with the normal composition, i.e., Zr_52.5_Cu_17.9_Al_10_Ni_14.6_Ti_5_(Vit105) and Zr_65_Cu_15_Ni_10_Al_10_ (in atomic percentage) were chose in this study. Master alloy ingots were prepared by arc melting mixed constitute elements with purity >99.5% in a Ti-getted atmosphere. Each alloy ingots were remelted at least three times for the compositional homogeneity. Rod-shape glassy samples with different diameters (2 and 3 mm) and a length of about 80 mm were obtained by suction copper mold casting. The amorphous nature of specimens were confirmed by the x-ray diffraction (XRD) method using a MAC Mo3 XHF diffractometer with *Cu Kα* radiation and the differential scanning calorimetry (DSC, Perkin Elmer DSC7).

For Cu electroplating, a common copper sulfate-sulfuric bath was used^31^. The solution used for electroplating was prepared from cupric sulfate pentahydrate and reagent 98 wt% pure grade sulfuric acid, from which an electrolyte with a concentration of 65 g/l copper ion and 73.5 g/l sulfuric acid. The electroplating was conducted at room temperature. The copper-phosphorous plate was used as the anode and a MG rod was used as the cathode. The distance between the anode and cathode was kept at about 30 mm. In the electrodeposition process, a current of density of 0.2 ± 0.01 mA/mm^2^ were applied for different plating for each experiment and the thickness of coating layers were controlled by tuning the plating time. In order to ensure that the thickness of the Cu coating was evenly distributed, the BMG rods were continuously rotated, driven by a motor at a constant speed of 50 rpm, in addition to the magnetic agitation. After electroplating, the thickness of the Cu coating was measured using optical microscopy (OM) equipped with length-scale measuring tools.

Samples with different diameters were cut from MG rods by a diamond saw with water cooling, and then carefully ground into compression specimens with an aspect ratio of 2:1 within an accuracy of ±5 μm at two ends. Uniaxial compression tests were performed with an Instron 556 electromechanical test system under the displacement-controlled mode. The load, the displacement and the time are recorded at a frequency of 500 Hz. The strain was measured by an extensometer which is attached to sample by a special setup. Special care was also taken to ensure that the two ends of test specimens were parallel and orthogonal to loading axis during tests. Each test at the same condition is performed at least 3 times, to obtain the reliability of the data. The deformation and fracture surface morphology of sample before and after mechanical tests were examined by a scanning electron microscopy (SEM, JEOL, JSM-5600).

## Additional Information

**How to cite this article**: Sun, B. A. *et al*. Origin of Shear Stability and Compressive Ductility Enhancement of Metallic Glasses by Metal Coating. *Sci. Rep*. **6**, 27852; doi: 10.1038/srep27852 (2016).

## Supplementary Material

Supplementary Information

## Figures and Tables

**Table 1:** The values of various parameters (sample diameter *D*, coating time, coating thickness, yield strength *σ*_*Y*_, plastic strain before fracture *ε*_*p*_, average shear slip size 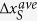, and maximum shear slip size 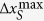) for different MG samples.

MGs	D (mm)	Coating time (min)	Coating Thickness (μm)	*σ*_*Y*_(*MPa*)	*ε*_*p*_(*%*)	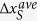 (μm)	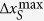 (μm)
Vit105	2	0	0	1736	3.95	2.39	5.32
Vit105	2	45	21.68	1708	14.77	1.36	5.40
Vit105	3	0	0	1755	0.34	–	–
Vit105	3	15	9.27	1726	4.21	2.08	5.73
Vit105	3	90	40.19	1697	3.58	1.64	5.81
Zr_65_Cu_15_Ni_10_Al_10_	2	0	0	1724	2.67	2.23	5.28
Zr_65_Cu_15_Ni_10_Al_10_	2	45	24.26	1701	4.07	2.01	5.39
Zr_65_Cu_15_Ni_10_Al_10_	3	0	0	1746	0.40	–	–
Zr_65_Cu_15_Ni_10_Al_10_	3	45	24.08	1706	6.22	2.07	5.84

## References

[b1] Schuh C., Hufnagel T. & Ramamurty U. Mechanical behavior of amorphous alloys. Acta Mater 55, 4067–4109 (2007).

[b2] Greer A. L., Cheng Y. Q. & Ma E. Shear bands in metallic glasses. Mater. Sci. Eng. R 74, 71–132 (2013).

[b3] Trexler M. M. & Thadhani N. N. Mechanical properties of bulk metallic glasses. Prog. Mater. Sci 55, 759–839 (2010).

[b4] Chen M. W. Mechanical Behavior of Metallic Glasses: Microscopic Understanding of Strength and Ductility. Annu Rev Mater Res 38, 445–469 (2008).

[b5] Spaepen F. A microscopic mechanism for steady state inhomogeneous flow in metallic glasses. Acta Metall 25, 407–415 (1977).

[b6] Song S. X. & Nieh T. G. Flow serration and shear-band viscosity during inhomogeneous deformation of a Zr-based bulk metallic glass. Intermetallics 17, 762–767 (2009).

[b7] Perepezko J. H., Imhoff S. D., Chen M.-W., Wang J.-Q. & Gonzalez S. Nucleation of shear bands in amorphous alloys. PNAS 111, 3938–3942 (2014). 24594599 10.1073/pnas.1321518111 PMC3964102

[b8] Liu Z. Y., Yang Y. & Liu C. T. Yielding and shear banding of metallic glasses. Acta Mater 61, 5928–5936 (2013).

[b9] Pan J., Chen Q., Liu L. & Li Y. Softening and dilatation in a single shear band. Acta Mater 59, 5146–5158 (2011).

[b10] Wang S. . Softening-induced plastic flow instability and indentation size effect in metallic glass. J Mech Phys Solids 77, 70–85 (2015).

[b11] Zhang Z. F., Eckert J. & Schultz L. Difference in compressive and tensile fracture mechanisms of Zr59Cu20Al10Ni8Ti3 bulk metallic glass. Acta Mater 51, 1167–1179 (2003).

[b12] Arora H. S., Aditya A. V. & Mukherjee S. Structural relaxation driven increase in elastic modulus for a bulk metallic glass. J App Phys 117, 014902 (2015).

[b13] Inoue A. & Takeuchi A. Recent development and application products of bulk glassy alloys. Acta Mater 59, 2243–2267 (2011).

[b14] Qiao J., Jia H. & Liaw P. K. Metallic glass matrix composites. Mater. Sci. Eng. R 100, 1–69 (2016).

[b15] Chen Y., Jiang M. Q. & Dai L. H. Collective evolution dynamics of multiple shear bands in bulk metallic glasses. Inter. J. Plast 50, 18–36 (2013).

[b16] Sun B. A. . Plasticity of Ductile Metallic Glasses: A Self-Organized Critical State. Phys Rev Lett 105, 035501 (2010). 20867777 10.1103/PhysRevLett.105.035501

[b17] Wang G. . Self-organized intermittent plastic flow in bulk metallic glasses. Acta Mater 57, 6146–6155 (2009).

[b18] Sun B. A. & Wang W. H. Fractal nature of multiple shear bands in severely deformed metallic glass. App. Phys. Lett 98, 201902 (2011).

[b19] Liu Y. H. . Super Plastic Bulk Metallic Glasses at Room Temperature. Science 315, 1385–1388 (2007). 17347434 10.1126/science.1136726

[b20] Chen L. Y. . New Class of Plastic Bulk Metallic Glass. Phys Rev Lett 100, 075501 (2008). 18352567 10.1103/PhysRevLett.100.075501

[b21] Schroers J. & Johnson W. L. Ductile Bulk Metallic Glass. Phys. Rev. Lett 93, 255506 (2004). 15697909 10.1103/PhysRevLett.93.255506

[b22] Hofmann D. C. . Designing metallic glass matrix composites with high toughness and tensile ductility. Nature 451, 1085–1089 (2008). 18305540 10.1038/nature06598

[b23] Pauly S., Gorantla S., Wang G., Kühn U. & Eckert J. Transformation-mediated ductility in CuZr-based bulk metallic glasses. Nat. Mater 9, 473–477 (2010). 20473286 10.1038/nmat2767

[b24] Wu Y., Xiao Y., Chen G., Liu C. T. & Lu Z. Bulk Metallic Glass Composites with Transformation-Mediated Work-Hardening and Ductility. Adv. Mater 22, 2770–2773 (2010). 20422654 10.1002/adma.201000482

[b25] Madge S., Louzguine-Luzgin D., Inoue A. & Greer A. Large Compressive Plasticity in a La-Based Glass-Crystal Composite. Metals 3, 41–48 (2012).

[b26] Zhang Y., Wang W. H. & Greer A. L. Making metallic glasses plastic by control of residual stress. Nat. Mater 5, 857–860 (2006). 17041581 10.1038/nmat1758

[b27] Qu R. T., Zhang Q. S. & Zhang Z. F. Achieving macroscopic tensile plasticity of monolithic bulk metallic glass by surface treatment. Scrip. Mater 68, 845–848 (2013).

[b28] Wang Q. . Superior Tensile Ductility in Bulk Metallic Glass with Gradient Amorphous Structure. Sci. Rep 4, 4757 (2014). 24755683 10.1038/srep04757 PMC3996486

[b29] Donohue A., Spaepen F., Hoagland R. G. & Misra A. Suppression of the shear band instability during plastic flow of nanometer-scale confined metallic glasses. App. Phys. Lett 91, 241905 (2007).

[b30] Wang Y., Li J., Hamza A. V. & Barbee T. W. Ductile crystalline–amorphous nanolaminates. PNAS 104, 11155–11160 (2007). 17592136 10.1073/pnas.0702344104 PMC1899185

[b31] Chen W., Chan K. C., Yu P. & Wang G. Encapsulated Zr-based bulk metallic glass with large plasticity. Mater. Sci. Eng. A 528, 2988–2994 (2011).

[b32] Yu P., Liu Y. H., Wang G., Bai H. Y. & Wang W. H. Enhance plasticity of bulk metallic glasses by geometric confinement. J. Mater. Res 22, 2384–2388 (2007).

[b33] Choi Y. C. & Hong S. I. Enhancement of plasticity in Zr-base bulk metallic glass by soft metal plating. Scrip. Mater 61, 481–484 (2009).

[b34] Chen W. . Plasticity enhancement of a Zr-based bulk metallic glass by an electroplated Cu/Ni bilayered coating. Mater. Sci. Eng. A 552, 199–203 (2012).

[b35] Qiu S.-B. & Yao K.-F. Novel application of the electrodeposition on bulk metallic glasses. App. Surf. Sci 255, 3454–3458 (2008).

[b36] Lu J. & Ravichandran G. Pressure-dependent flow behavior of Zr41.2Ti13.8Cu12.5Ni10Be22.5 bulk metallic glass. J. Mater. Res 18, 2039–2049 (2003).

[b37] Kanani N. Electroplating and Electroless Plating of Copper and its Alloys. (Finishing Publications Ltd., 2003).

[b38] Wright W. J., Schwarz R. B. & Nix W. D. Localized heating during serrated plastic flow in bulk metallic glasses. Mater. Sci. Eng. A 319–321, 229–232 (2001).

[b39] Dalla Torre F. H., Klaumünzer D., Maaβ R. & Löffler J. F. Stick–slip behavior of serrated flow during inhomogeneous deformation of bulk metallic glasses. Acta Mater 58, 3742–3750 (2010).

[b40] Sun B. A. . Serrated flow and stick–slip deformation dynamics in the presence of shear-band interactions for a Zr-based metallic glass. Acta Mater 60, 4160–4171 (2012).

[b41] Ke H. B., Sun B. A., Liu C. T. & Yang Y. Effect of size and base-element on the jerky flow dynamics in metallic glass. Acta Mater 63, 180–190 (2014).

[b42] Tong X. . Shear avalanches in plastic deformation of a metallic glass composite. Inter. J. Plast 77, 141–155 (2016).

[b43] Sun B. A. . Origin of Intermittent Plastic Flow and Instability of Shear Band Sliding in Bulk Metallic Glasses. Phys. Rev. Lett 110, 225501 (2013). 23767733 10.1103/PhysRevLett.110.225501

[b44] Cheng Y. Q., Han Z., Li Y. & Ma E. Cold versus hot shear banding in bulk metallic glass. Phys. Rev. B 80, 134115 (2009).

[b45] Han Z., Wu W. F., Li Y., Wei Y. J. & Gao H. J. An instability index of shear band for plasticity in metallic glasses. Acta Mater 57, 1367–1372 (2009).

[b46] Furukawa A. & Tanaka H. Inhomogeneous flow and fracture of glassy materials. Nat. Mater 8, 601–609 (2009). 19525951 10.1038/nmat2468

[b47] Sarmah R., Ananthakrishna G., Sun B. A. & Wang W. H. Hidden order in serrated flow of metallic glasses. Acta Mater 59, 4482–4493 (2011).

[b48] Kuzmin O. V., Pei Y. T. & De Hosson J. T. M. Size effects and ductility of Al-based metallic glass. Scrip. Mater 67, 344–347 (2012).

[b49] Ren L. W. . Enhancement of plasticity in Zr-based bulk metallic glasses electroplated with copper coatings. Intermetallics 57, 121–126 (2015).

[b50] Chen C. Q., Pei Y. T. & De Hosson J. T. M. Effects of size on the mechanical response of metallic glasses investigated through *in situ* TEM bending and compression experiments. Acta Mater 58, 189–200 (2010).

[b51] Chen C. Q. . Intrinsic size effects in the mechanical response of taper-free nanopillars of metallic glass. Phys. Rev. B 83, 180201 (2011).

[b52] Kuzmin O. V., Pei Y. T., Chen C. Q. & De Hosson J. T. M. Intrinsic and extrinsic size effects in the deformation of metallic glass nanopillars. Acta Mater 60, 889–898 (2012).

