# Supplementary material for: Origin of Shear Stability and Compressive Ductility Enhancement of Metallic Glasses by Metal Coating
Source: Sci Rep. 2016 Jun 8;6:27852. doi: 10.1038/srep27852 (PMC4897694; doi:10.1038/srep27852)
Supplement: Supplementary Information [file srep27852-s1.pdf]

Supplementary Materials For

Origin of Shear Stability and Compressive Ductility Enhancement of Metallic Glasses by Metal Coating

B. A. Sun[a], S. H. Chen[b], Y.M. Lu[a], Z. G. Zhu[c], Y. L. Zhao[a], Y. Yang[a], K. C. Chan[b], C. T. Liu[a,]*

[a]Centre For Advanced Structural Materials, Department of Mechanical Biomedical Engineering, City University of Hong Kong, Hong Kong

[b]Advanced Manufacturing Technology Research Centre, Department of Industrial and Systems Engineering, The Hong Kong Polytechnic University, Kowloon, Hong Kong.

[c]Department of Physics and Materials Science, City University of Hong Kong, Hong Kong

***To whom correspondence may be addressed*: chainliu@cityu.edu.hk (C. T. Liu)**

Supplementary Note I. Mechanistic analysis on the interplay between the coating layer and shear deformation in MGs

The confinement of the coating layer on the deformation of MGs is schematically shown in Fig.8. Obviously, once a shear step slides out of the surface of MGs by shear banding, the coating layer ring near the shear step will be subjected to a uniform tension stress, T_1, along the tangential direction of the coating ring, due to the increase of circumferential length (see Fig.8b). From the geometrical relation shown in Fig.8b, T_1 for the elastic deformation of Cu-layer can be simply calculated by

$$T_1 = E^{Cu}\Delta\varepsilon = 4hE^{Cu} / \pi D \quad \text{(S1)}$$

where E^{Cu} is the Young's modulus of electrodeposited Cu-layer, h is the height of surface shear step and D is the sample diameter. In general, E^{Cu}~120 GPa, and h~3-5 μm for a typical shearing event, thus, T_1 could easily reach the yield strength of Cu-layer (~120-150 MPa)[1] in a shearing event, and after the yielding, T_1 increases slowly until reach the final fracture strength of Cu-layer, σ_F^{Cu} (~200-300 MPa), after then the Cu-layer will fracture with crack formation along the sample lateral side, as what is happened in final deformation stage of coated 2-mm MG samples. Conversely, the coating layer exerts a radial pressure, p, on the MG and also a force, F_{y1}, to resist to the shear-step sliding. It is easily shown that

$$p = 2T_1\delta / D, \quad F_{y1} = 2T_1\delta h \cot\theta \quad \text{(S2)}$$

where δ is the coating-layer thickness, θ is the shear angle and $h\cot\theta$ can be regarded as the lateral length of the coating-lay ring subjected to tension.

In addition to the circumferential tension, the shear step sliding out of MG

surface also exert an tensile force on the coating layer along the lateral direction, as shown schematically in Fig.7(c). The tension of the coating layer will cause interface debonding along lateral direction, which should be the main reason for the formation of circumferential bulge, as observed in experiments (Fig.6(c)). Due to the bending of coating layer, the tension stress in the coating layer is different in the CD and DE part, and is denoted as T_2 and T_3, respectively.With force balance, we can easily obtain that $T_3 = lT_2 / \sqrt{h^2 + l^2}$, where l is lateral length projected by the bent part of coating layer and (l+ $hcot\theta$) can be regarded as the debonding length. Similar to the case of circumferential tension, T_2 and T_3 initially increase with h (for a certain l) and quickly reach the yield strength of Cu-layer and after then, increase slowly until T_2 reach tensile fracture strength σ_F^{Cu} , where the fracture of coating layer occurs. The lateral tension of coating layer also results in a transverse force F_{y2}, resisting to the shear band sliding. If we assume that the debonding is circumferential, the F_{y2} is related with T_3 by:

$$F_{y2} = \pi D T_3 h\delta / l \qquad \text{(S3)}$$

From Eq.S3, one can see that F_{y2} depends on the value of l, and can Shave a large value if l is small, which may play an important role in retarding shear band dynamics.

Supplementary Note II. The quantitative analysis on the interface debonding

With the increase of h, debonding of the coating layer along sample lateral may also occurs, which release the elastic energy stored in the coating layer and result in the

increase of *l*. For a certain *h*, the debonding occurs for $-dU_e / dl \geq \Gamma_i$, where U_e is the stored elastic energy per unit circumferential length, Γ_i is the interface toughness for the coating Cu-layer[2]. Given *h* and *l*, U_e can be expressed as:

$$U_e = \frac{T_2^2}{2E_{Cu}} \delta \sqrt{h^2 + l^2} + \frac{T_3^2}{2E_{Cu}} \delta h \cot\theta \qquad \text{(S4)}$$

Since $T_2 = T_3 \sqrt{h^2 + l^2} / l$, taking this into Eq.S4 and considering T_3 (roughly equal the yielding stress of Cu-layer) is weakly dependent on *h* and *l*, the interface debonding occurs for:

$$-\frac{dU_e}{dl} = \frac{T_3^2 \delta}{2E} \left[2(h/l)^2 - 1 \right] \sqrt{(h/l)^2 + 1} \geq \Gamma_i \qquad \text{(S5)}$$

From Eq.S5, we can see that the interface debonding is mainly determined the ratio *h/l* and Γ_i. And the necessary or the minimum condition for the debonding is $2(h/l)^2 - 1 > 0$, i.e., $h/l > \sqrt{2}/2$. From Eq.S5, a critical value *h/l*, λ_c, can also be determined by solving

$$f(h/l) = -dU_e / dl - \Gamma_i = 0 \qquad \text{(S6)}$$

Given a certain *h*, debonding occurs for $l<l_c=h/\lambda_c$. Similarly, for a certain *l*, debonding occurs for $h>h_c=l\lambda_c$. In this sense, the layer with the larger thickness are more susceptible to debonding as the larger δ results in the smaller λ_c provided other parameters are unchanged. At the beginning when the shear step is just slides out of surface, $l<<h$ and *h/l* should be much larger than λ_c, interface debonding has to occur to release the stored elastic energy in the coating layer, otherwise, the tension stress T_2 will be so large to cause the fracture of the coating layer. However, once a stable length *l* is created, the coating layer could either be deformed with increase of the

tension stress until the final fracture or debond further with the increase of l and meanwhile maintaining or decreasing the tensile stress, which depends on the specific coating quality or interface toughness. The lower Γ_i often leads to debonding rather than fracture of the coating layer, which will not be effective to resist to shear band sliding.

References

1. Chen W, Chan KC, Chen SH, Guo SF, Li WH, Wang G. Plasticity enhancement of a Zr-based bulk metallic glass by an electroplated Cu/Ni bilayered coating. *Mater Sci Eng A* 2012, **552:** 199-203.

2. Hojo M, Tanie Y, Sugano M, Inoue Y, Nishikawa M, Shikimachi K*, et al.* Mode I type interlaminar fracture toughness of Cu plated Gd-YBCO coated conductor. *Physics Procedia* 2012, **27:** 252-255.
